# Delayed Disease in Cynomolgus Macaques Exposed to Ebola Virus by an Intranasal Route

**DOI:** 10.3389/fimmu.2021.709772

**Published:** 2021-08-16

**Authors:** Sara C. Johnston, Catherine L. Wilhelmsen, Joshua Shamblin, Adrienne Kimmel, Justine Zelko, Suzanne Wollen, Arthur J. Goff

**Affiliations:** ^1^Virology Division, United States Army Medical Research Institute of Infectious Diseases, Fort Detrick, MD, United States; ^2^Pathology Division, United States Army Medical Research Institute of Infectious Diseases, Fort Detrick, MD, United States; ^3^Veterinary Medicine Division, United States Army Medical Research Institute of Infectious Diseases, Fort Detrick, MD, United States; ^4^Research Program Office, United States Army Medical Research Institute of Infectious Diseases, Fort Detrick, MD, United States

**Keywords:** Ebola, EBOV, macaque, NHP, mucosal, intranasal, animal model

## Abstract

Ebola virus remains a significant public health concern due to high morbidity and mortality rates during recurrent outbreaks in endemic areas. Therefore, the development of countermeasures against Ebola virus remains a high priority, and requires the availability of appropriate animal models for efficacy evaluations. The most commonly used nonhuman primate models for efficacy evaluations against Ebola virus utilize the intramuscular or aerosol route of exposure. Although clinical disease signs are similar to human cases, disease progression in these models is much more rapid, and this can pose significant hurdles for countermeasure evaluations. The objective of the present study was to evaluate the Ebola virus disease course that arises after cynomolgus macaques are exposed to Ebola virus by a mucosal route (the intranasal route). Two different doses (10 pfu and 100 pfu) and delivery methodologies (drop-wise and mucosal atomization device) were evaluated on this study. Differences in clinical disease between dose and delivery groups were not noted. However, a delayed disease course was identified for approximately half of the animals on study, and this delayed disease was dose and administration method independent. Therefore, it appears that mucosal exposure with Ebola virus results in a disease course in cynomolgus macaques that more accurately replicates that which is documented for human cases. In summary, the data presented support the need for further development of this model as a possible alternative to parenteral and small-particle aerosol models for the study of human Ebola virus disease and for countermeasure evaluations.

## Introduction

Ebola virus (EBOV), and the closely related Marburg virus, is a filovirus that causes severe and often lethal hemorrhagic fever disease in humans and nonhuman primates. The development of vaccines and other medical countermeasures against EBOV remains a high priority, and current approaches to evaluating countermeasures have utilized the US Food and Drug Administration (FDA) Animal Rule guidance wherein the FDA may grant approval for medical countermeasures based on animal studies when evaluation of efficacy in humans is not ethical or feasible. The animal model to be used for such studies should be relevant to the human disease and relevant to the countermeasure’s mechanism of action to assure findings in the animal model adequately predict efficacy in humans.

The most commonly used animal models for efficacy evaluations against filoviruses have been nonhuman primates (NHP) (i.e. cynomolgus and rhesus macaques). Intramuscular (IM) virus inoculations, which represent exposure *via* a needle stick or bite injury, have been frequently used for countermeasure studies in NHP, with small particle aerosol (AE) exposures used mainly for efficacy evaluations for biodefense purposes. For both inoculation strategies, clinical disease signs are similar to human cases. However, disease progression is very rapid, with death occurring within 6-9 days of exposure (1, 2), compared to 6-16 days following symptom onset in humans (3) (information also available on the World Health Organization fact sheets on EBOV and Marburg virus). The incubation period is also typically shorter for primates (6-7 days) compared to humans (2-21 days) (1, 2) (information also available on the World Health Organization fact sheets on EBOV and Marburg virus). These differences in disease course generally do not affect vaccine evaluations wherein protective immunity should be present long before the animal is exposed to virus. However, rapid disease progression can pose significant issues for drug evaluations. This is particularly problematic for therapeutic indications where treatment may not be initiated until a relevant “trigger” is reached. This trigger, usually based on clinical disease presentation for a human patient, is often fever and/or the presence of viral RNA in the blood. For most primates infected with filoviruses, the time from trigger to death is usually extremely short (1-5 days, depending on the trigger used), whereas for humans it can be up to 16 days. This means that a drug will generally have significantly longer to mitigate the virus in a human patient compared to in a primate, so a lack of efficacy for this indication in a primate may not necessarily mean a lack of efficacy in a human patient. Therefore, current NHP models may not be ideal to allow extrapolation of efficacy in humans for certain medical countermeasures; thus, other options should continue to be explored.

The other issue with current NHP models is they use routes of exposure that do not mimic the most common natural disease transmission route, which is predicted to be mucosal (4–6). From a natural infection perspective, an intranasal (IN) inoculation strategy is relevant. Based on EBOV studies conducted in cynomolgus macaques (CM), clinical disease signs are similar following IN inoculation compared to IM and aerosol inoculation, and mimic disease characteristics noted in human cases (7, 8). Based on these data, IN exposure of NHP for filovirus studies warranted further investigation as these models may be ideal candidates for countermeasure testing as disease progression is more relevant to human cases, compared to the established IM and AE models. To that end, in the present study we sought to further characterize the IN EBOV model in CM. CMs were randomly assigned to one of four groups and exposed on Study Day 0 to a target dose of 10 or 100 plaque forming units (pfu) of EBOV using a drop-wise method or a mucosal atomization device (MAD). Daily observations of the animals were performed and clinical signs of disease recorded. Blood was collected at multiple time points throughout the study for analysis of hematology, serum chemistries, coagulation, and quantitative real-time polymerase chain reaction (qRT-PCR) for viral RNA in plasma. Animals developed clinical and hematological signs of EBOV disease consistent with historical data in CM (9–12) and human cases (13, 14) and, in general, statistically-relevant differences between groups were not noted. Delayed disease onset was noted for approximately half of the animals on study, including approximately half of the animals in each group, and one animal in the 10 pfu MAD group survived until end-of-study (Study Day 28). The data, therefore, support the conclusion that there were two distinct critical phases on this study, suggesting that a disease course more reminiscent of human cases is achievable using this EBOV model.

## Materials and Methods

### Animals

Animal research was conducted at the United States Army Medical Research Institute of Infectious Diseases (USAMRIID). Twenty-one adult *Macaca fascicularis* (cynomolgus macaques, CM) of Chinese origin, mixed male and female, were included on this study. At the time of challenge, animals were between 5-10 years old, and weighed between 3.8-7.8 kg; group mean ages and body weights were similar. All animals had passed a semi-annual physical examination and were certified as healthy by a veterinarian, and all animals were serologically naïve for prior filovirus infection. Animals were acclimated in ABSL-4 animal rooms for 7 days prior to virus exposure (i.e., Study Day 0) and housed individually in 4.3 square foot cages. Animal rooms were lit with fluorescent lights and maintained on a 12-hour light/dark cycle, except when interrupted by study events during the *in-life* portion of the study, animals were provided Teklad Global 20% Protein Primate Diet (2050C) (Harlan Teklad, Frederick, MD), fruits, and water *ad libitum via* an automatic watering system, and animals were given enrichment regularly as recommended by the Guide for the Care and Use of Laboratory Animals. In addition, oral rehydration solution (i.e., Pedialyte^®^) was provided to all animals when one or more animals displayed signs of illness and was discontinued when all animals were deemed recovered. Procedures involving manipulation of the animals, including phlebotomy and physical examination, were performed while the animals were under anesthesia.

#### Ethics Statement

These experiments and procedures were reviewed and approved by the United States Army Medical Research Institute for Infectious Diseases Institutional Animal Care and Use Committee. All research was conducted in compliance with the United States Department of Agriculture Animal Welfare Act (PHS Policy) and other federal statutes and regulations relating to animals and experiments involving animals, and adheres to the principles stated in the Guide for the Care and Use of Laboratory Animals, National Research Council, 2011. The facility is fully accredited by the Association for Assessment and Accreditation of Laboratory Animal Care, International. The animals were provided food and water ad libitum and checked at least daily according to the protocol. All efforts were made to minimize painful procedures; the attending veterinarian was consulted regarding painful procedures, and animals were anesthetized prior to phlebotomy and virus infection. Animals were humanely euthanized at the end of study by intracardiac administration of a pentobarbital-based euthanasia solution under deep anesthesia in accordance with current American Veterinary Medical Association Guidelines on Euthanasia and institute standard operating procedures.

### Virus and Virus Exposure

Ebola virus (EBOV) (designated 199510621, GenBank Accession Number AY354458.1) was obtained from a lethal case during an outbreak in the Democratic Republic of the Congo (formerly Zaire) in 1995. The first passage of virus, designated virus seed pool 807223, was conducted at the Centers for Disease Control and Prevention using Vero E6 cells. A second passage of virus, designated WRC000121, was conducted at the University of Texas Medical Branch. WRC000121 was transferred to USAMRIID and propagated on BEI Resources (Manassas, VA) Vero E6 cells to produce the USAMRIID master seed stock, R4415, a 7U variant (92.8% of the 7U variant). This stock was fully sequenced and tested for sterility; no known contaminants were detected in the stock. The stock has a certified titer of 1.31x10^6^ pfu/mL as determined by agarose plaque titration assay. On Study Day 0, animals were exposed to a target dose of either 10 or 100 pfu of EBOV by the IN route; delivery was either drop-wise using a pipette or was administered *via* MAD in a total volume of 1 mL (0.5 mL delivered to each nare). A sample of the material used for administration for each dose group was titrated by plaque assay to determine the delivered pfu.

### Animal Observations

Animals were observed cage side daily for signs of clinical disease including, but not limited to, the following: responsiveness, cough, edema, rash, bleeding, and motor function. Other observations (such as, biscuit/fruit consumption, condition of stool, and urine output) were documented when possible. Observations were increased to up to four times daily (4-6 hours apart) when clinical signs of illness were noted. Observations under anesthesia (i.e. physical examinations) occurred after cage side observations on Study Days 0, 3, 6, 10, 14, and 21 (based on survival), and on the day of death/euthanasia. Physical examinations included the following: determination of body weight, determination of rectal temperature, blood collection, and clinical observations for signs of illness (including, but not limited to, rash, bleeding, exudate, edema, lymphadenopathy, and oral abnormalities).

Animals were humanely euthanized under deep anesthesia by barbiturate overdose at end-of-study or when the following criteria were met (defined as moribund):

Responsiveness Score = 4 (persistently prostrate, severely or completely unresponsive, may have signs of respiratory distress); OR
Responsiveness Score = 3 (prostrate but able to rise if stimulated or moderate to dramatically reduced to response to external stimuli) and rectal temperature ≤34°C; OR
Responsiveness Score = 3 and two or more chemistry values outside of range [Blood urea nitrogen (BUN) ≥ 68 mg/dL, calcium (CA) ≤ 6.8 mg/dL, gamma-glutamyl transferase (GGT) ≥ 391 U/L, and/or creatinine (CRE) ≥ 2.8 mg/dL]

### Necropsy, Histology, and Immunohistochemistry

Necropsies were conducted by a veterinary pathologist on all animals in this study. Necropsies were performed as soon as feasible after notification of a euthanasia or an animal was found deceased, typically within 8 hours. Animal carcasses were held in a carcass refrigerator while awaiting necropsy to maintain tissue integrity. Tissues were collected and fixed by immersion in containers of 10% neutral buffered formalin for 21 days prior to further processing.

The tissue samples were trimmed, routinely processed, and embedded in paraffin. Sections of the paraffin-embedded tissues 5 um thick were cut for histology. For histology, slides were deparaffined, stained with hematoxylin and eosin, coverslipped, and labeled. Replicate tissue sections were placed on positive-charged slides and stained for immunohistochemistry using a cocktail of mouse monoclonal anti-EBOV antibody.

### Clinical Pathology

Blood for clinical pathology was collected on Study Days -13, 0, 3, 6, 10, 14, 21, and the day of euthanasia (either moribund or end-of-study). For serum chemistries, whole blood was collected into Serum Clot Activator Greiner Vacuette tubes (Greiner Bio-One, Monroe, NC). Tubes were allowed to clot for at least 10 min and the serum separated in a centrifuge set at 1800 × g for 10 min at ambient temperature. The required volume of serum was removed for chemistry analysis using a General Chemistry 13 panel (Abaxis, Union City, CA) on a Piccolo Point-Of-Care Analyzer (Abaxis, Union City, CA). Serum was removed from the clot within 1 hour of centrifugation and was analyzed within 12 hours of collection.

For hematology, whole blood was collected into Greiner Vacuette blood tubes containing K3 EDTA as an anti-coagulant. Hematology was performed on an ADVIA 120 System (Siemens Healthineers, Munich, Germany) within 8 hours of collection to obtain accurate differential counts. Plasma was then separated in a centrifuge set at 2500 × g for 10 min at ambient temperature.

For coagulation, whole blood was collected into Greiner Vacuette blood tubes containing sodium citrate. Plasma was separated within 4 hours of collection in a centrifuge set at 1500 × g for 10 min at ambient temperature. The required volume of plasma was removed for coagulation analysis on a Sysmex^®^ CA-1500 System (Siemens Healthineers, Munich, Germany).

### qRT-PCR

A 100 uL volume of EDTA plasma was added to 300 uL of TRIzol^®^ LS (Thermo Fisher Scientific, Waltham, MA) for RNA isolation for qRT-PCR. Samples were extracted and eluted with AVE buffer using a QIAamp^®^ Viral RNA Mini Kit (Qiagen, Germantown, MD). All samples were used in qRT-PCR on an Applied Biosystems™ 7500 Fast Dx instrument (Applied Biosystems, Foster City, CA) using the following thermal cycling conditions: reverse transcription for 15 min at 50°C; Taq activation for 5 min at 95°C; 45 cycles of denature (1 sec at 95°C) and anneal/extend (26 sec at 60°C). The RT-PCR reaction used the Invitrogen™ SuperScript II^®^ One-Step RT-PCR System (Invitrogen, Carlsbad, CA) with additional MgSO_4_ added to a final concentration of 3.0 mM. The sequence of the primer and probes for the EBOV nucleoprotein gene are described below. The genomic equivalents (ge) were determined using a standard curve of synthetic RNA of known concentration.

Forward primer (1 µM): 5′ - TTT TCA ATC CTC AAC CGT AAG GC - 3′

Reverse primer (1 µM): 5′ - CAG TCC GGT CCC AGA ATG TG - 3′

Probe (0.1 µM): 6FAM - CAT GTG CCG CCC CAT CGC TGC – TAMRA

### Statistical Analyses

Analysis was performed using SAS Version 9.41. The data were imported into SAS using SAS PC Files Server. Three animals who were identified as positive for Mycobacterium tuberculosis were excluded from analysis. Animals that survived to 28 days after challenge were considered survivors and were censored to date of last observation. Exploratory subgroup analysis was performed comparing animals with early/normal days of death (≤ 10 days post-challenge) to surviving animals/animals with delayed days of death (>10 days post-challenge). The mean time to death following challenge for each group and subgroup was calculated. Rate of survival was calculated using the Kaplan-Meier method2 and compared between groups and subgroups by log-rank tests. Wilcoxon rank-sum tests3 were used to compare disease manifestations between subgroups on study days -13, 0, 3, 6, and 9/10. Data for Study Days 13/14 were available only for the later death subgroup; therefore, no comparative analyses were conducted for that time point. Comparisons between subgroups were also made using each animal’s last observation prior to death or end of study. Assay values below the lower limit of detection were set to a value equal to the lower limit of detection divided by the square root of 2 (LLOD/√2) prior to analysis. Assay values above the upper limit of detection were set to a value equal to the upper limit of detection prior to analysis. Missing data were handled as missing at random, and no corrections for missing data were included in the analysis. All significance tests performed were two-tailed. Significance levels were set at α = 0.05. As the subgroup analysis was exploratory, no corrections for multiple pairwise comparisons were made. Descriptive statistics including number of observations, mean, standard deviation, median, minimum and maximum were calculated.

## Results

### Clinical Disease Following Intranasal Exposure to EBOV

Twenty-one CM were randomly assigned to four groups (**Table 1**), using gender, weight and age as stratification parameters to achieve similar group means and genders. Group 1 animals (CMs 1-6) and Group 2 animals (CMs 7-12) were exposed to 7 pfu EBOV, and Group 3 animals (CMs 13-17) and Group 4 animals (CMs 18-21) were exposed to 125 pfu EBOV. For intranasal administration, a drop-wise technique was used for Groups 1 and 3, and MADs were used for Groups 2 and 4. MADs are designed to create a fine mist aerosol containing particles 30-100 µm in size. All virus exposures occurred on Study Day 0. Animals were monitored daily for clinical signs of disease, and were euthanized when they reached a terminal disease state. One animal in Group 2 survived to end-of-study (CM 11) and was euthanized on Study Day 28. For all analyses to be described herein, animal results are discussed by study group (Group 1, 2, 3, or 4), and/or by the time point that the animal was euthanized/succumbed [early-phase animals (referring to animals that were euthanized/succumbed during a typical EBOV window for CMs of Study Days 5 through 10) or late-phase animals (referring to animals that were euthanized/succumbed between Study Days 11 and 18)]. Survivor refers to the only animal that survived until the end of the study, CM 11.

**Table 1:** Study Design.

Group	Total # NHP (Early-Phase/Late Phase/Survivors)	Exposure route (Study Day 0)	Target exposure dose (pfu)	Actual exposure dose (pfu)
1	6 (3/3/0)	IN, Drop-wise	10	7
2	6 (1/4/1)	IN, MAD	10	7
3	5 (2/3/0)	IN, Drop-wise	100	125
4	4 (2/2/0)	IN, MAD	100	125

IN, intranasal; MAD, mucosal atomization device; pfu, plaque forming units.

**Figure 1:**
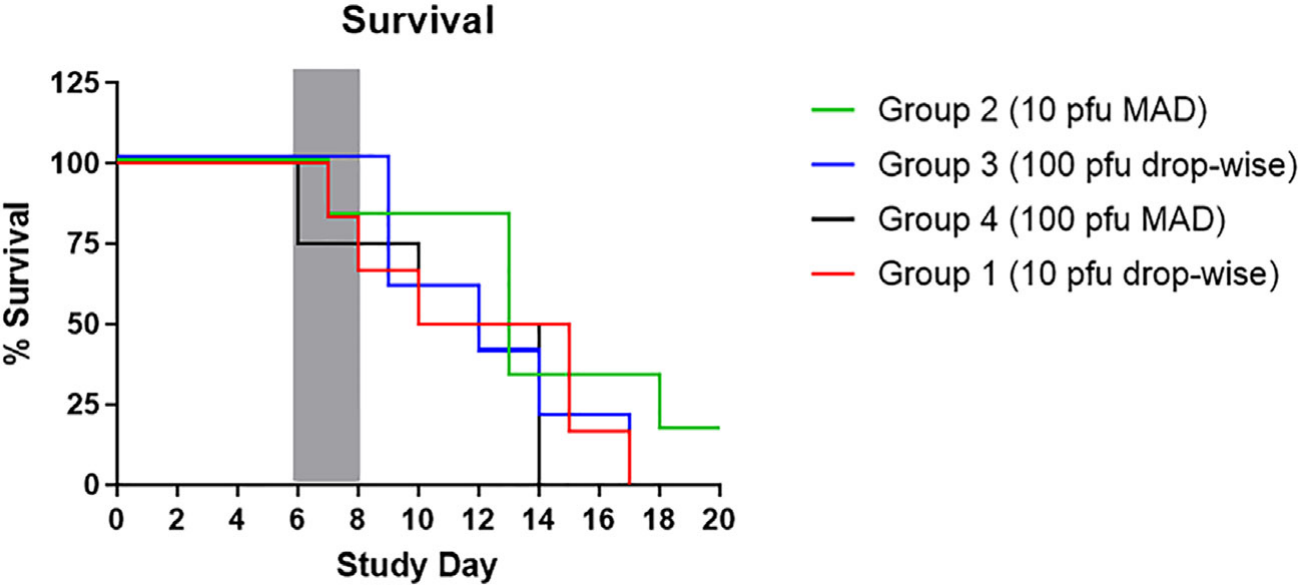
Kaplan-Meier Survival Curve. The figure shows the percent survival by study group. The grey box indicated the typical death window for CM infected with 100 pfu of EBOV by the IM or AE routes.

The Kaplan-Meier survival curve can be seen in **Figure 1**. The mean time to death for animals on study (excluding CM 11) was 11.13-12.97 days. Comparison of Kaplan-Meier survival curves by log-rank test indicated there were no statistically significant differences between the four study groups in terms of survival. However, a trend toward longer survival for Group 2 animals was observed. For this group, all but one animal was a late-phase animal or the end-of-study survivor, whereas the proportion of early and late phase animals was nearly identical for the other study groups. Correlations between early and late-phase animals were also examined. Early-phase animals had a mean time to death of 8.34 days, compared to late-phase animals that had a mean time to death of 14.67 days. This significant difference indicates a delayed disease for 60 percent of the animals on this study.

**Figure 2:**
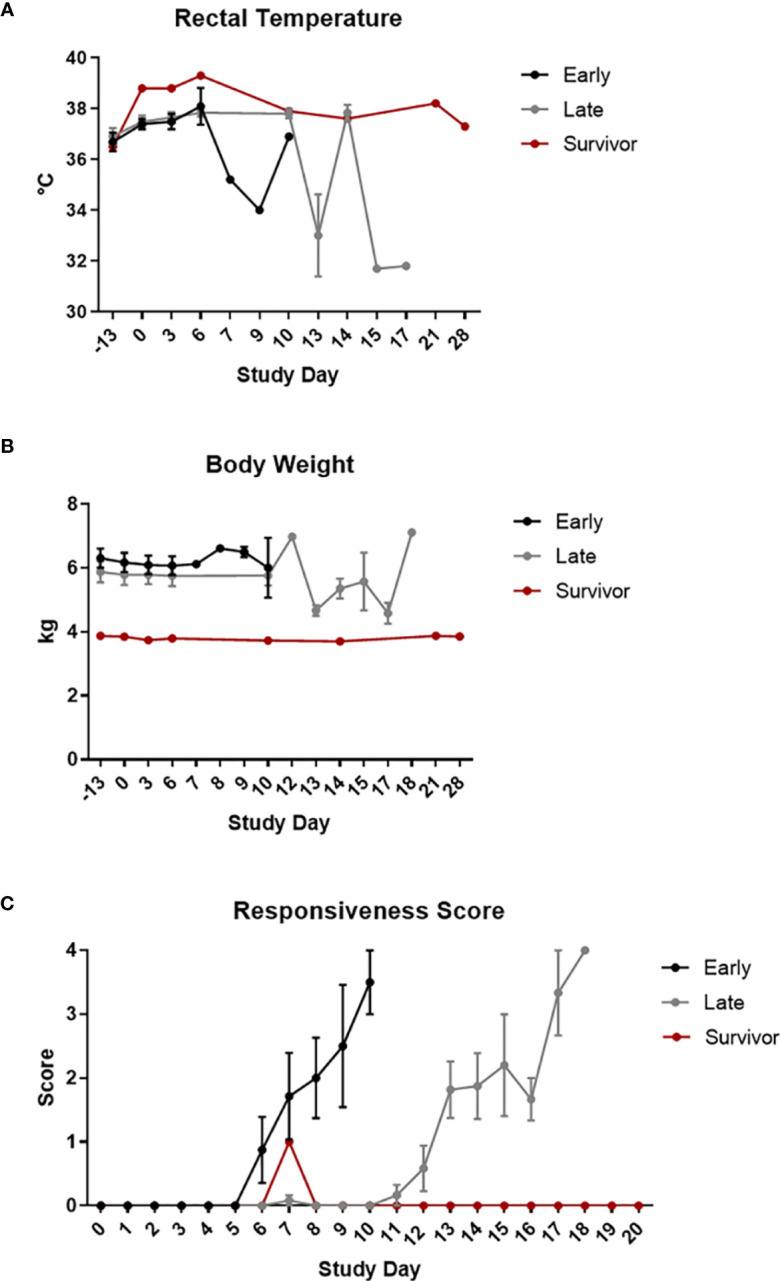
Clinical Disease. Rectal temperatures **(A)**, body weights **(B)**, and responsiveness scores **(C)** are shown grouped by survival phase (i.e., early-phase, late-phase, and survivor). Error bars represent the standard error measurement (SEM).

Regardless of group, animals that did not survive exhibited typical signs of EBOV disease, including decreased responsiveness, fever (defined as a rectal temperature greater than or equal to 1.5°C above baseline, with baseline being the mean of data from Study Day -13 and Study Day 0 for a particular animal), weight loss (loss of greater than 0.2 kg compared to baseline), rash, lymphadenopathy, anorexia, motor dysfunction, and terminal hypothermia (defined as a rectal temperature greater than or equal to 2°C below baseline) (**Table 2** and **Figure 2**).

**Table 2:** Summary of clinical disease findings.

CM #	1	2	3	4	5	6	7	8	9	10	11	12	13	14	15	16	17	18	19	20	21
Group	1	1	1	1	1	1	2	2	2	2	2	2	3	3	3	3	3	4	4	4	4
Phase	Early	Early	Late	Early	Late	Late	Early	Late	Late	Late	EOS	Late	Late	Early	Late	Early	Late	Late	Early	Late	Early
Fever^1^ (Day of Onset)		X (6)	X (14)		X (14)		X (6)				X (6)			X (6)	X (10)			X (14)			X (6)
Weight Loss^2^	X	X	X		X		X	X	X			X			X	X		X		X	
Hypothermia^3^			X		X			X	X							X			X		
Moderately or Dramatically Reduced Responsiveness		X	X	X	X	X	X	X	X	X		X	X	X	X	X	X	X	X	X	X
Skin Rash	X	X	X	X	X	X	X	X	X	X		X	X	X	X	X	X	X	X	X	X
Lymphadenopathy			X		X	X		X	X	X	X		X		X	X	X	X	X		X
Facial/Mouth Bleeding				X																	X
Rectal Bleeding									X												
Pale Gums	X										X						X			X	X
Facial Swelling						X						X									
Motor Dysfunction		X	X	X	X	X	X	X		X		X		X		X			X	X	
Anorexia^4^	X	X	X	X	X	X						X	X	X	X	X	X	X		X	X
Cough																				X	
Icterus			X									X									X
Vomit				X*		X			X												
Nasal/Oral Discharge															X					X	
No Stool Present		X	X	X	X	X		X				X	X	X			X	X		X	X
Stool Not Fully Formed/Liquid								X				X								X	X

^1^Defined as a rectal temperature greater than or equal to 1.5°C above baseline, with baseline being the mean of data from Study Days -13 and Study Day 0 for a particular animal.

^2^Loss of greater than 0.2 kg compared to baseline.

^3^Defined as a rectal temperature greater than or equal to 2°C below baseline.

^4^Defined as an absence of biscuit and enrichment consumption for one or more days OR an absence of biscuit consumption for 3 or more consecutive days.

*Black vomit.

EOS, end of study (survivor).

Responsiveness was measured as a score from 0-4. A score of 0 was given to an NHP that was alert, responsive, exhibited normal activity, and was free of disease signs or exhibited only resolved/resolving disease signs. A score of 1 was given to an NHP that had slightly diminished general activity, and was subdued but responded normally to external stimuli. A score of 2 was given to an NHP that was withdrawn, may have had its head down, was exhibiting a fetal posture, was hunched, and had reduced response to external stimuli. A score of 3 was given to an NHP that was prostrate but able to rise if stimulated, or had moderate to dramatically reduced response to external stimuli. A score of 4 was given to an NHP that was persistently prostrate, or was severely or completely unresponsive, and may have had signs of respiratory distress; this score was also applied to animals found dead for analysis purposes. In general, and regardless of study group or phase, animals that succumbed rapidly progressed from a score of 0 to a score of 4.

Although fever was noted for less than half of the animals on this study, it is predicted that the observed occurrence was lower than the actual occurrence and resulted from multiple day stretches between collection events. Results of Wilcoxon rank-sum tests showed significant differences in temperature on Study Day 6 between the early-phase animals and late-phase animals, with temperature being significantly higher in the early-phase animals than in the late-phase animals. Other less common findings included bleeding, icterus, pale gums, vomit (including “black” vomit characteristic of advanced EBOV disease), loose/liquid stool, and facial swelling. Cough was noted for CM 20.

The majority of the animals on this study that lost weight were late-phase animals. Results of Wilcoxon rank-sum tests showed no significant differences in body weight between the early-phase animals and the late-phase animals.

There was a delay in the development of clinical signs for late-phase animals compared to historical controls and early-phase animals, with late-phase animals developing clinical signs on or after Study Day 10.

The survivor had relatively few clinical signs of EBOV disease that included fever, lymphadenopathy, and pale gums noted between Study Days 6-10.

### Clinical Pathology Analyses

**Figure 3:**
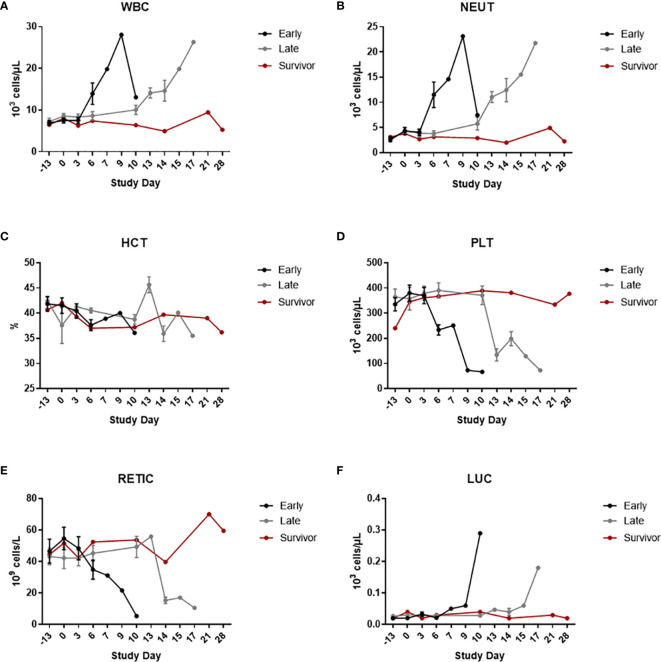
Hematology. Hematology was measured using an ADVIA 120 Hematology System. The graphs in this figure show data grouped by survival phase (i.e., early-phase, late-phase, and survivor). **(A)** WBC; **(B)** NEUT; **(C)** HCT; **(D)** PLT; **(E)** RETIC; **(F)** LUC. Error bars represent the SEM.

**Figure 4:**
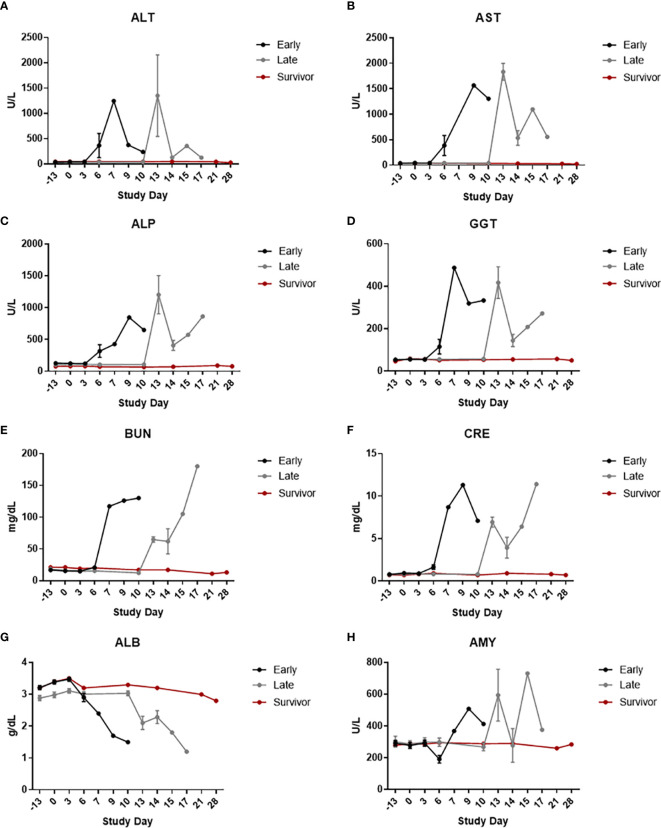
Clinical Chemistries. Serum clinical chemistry data was generated using Piccolo Point-Of-Care instruments. The graphs in this figure show data grouped by survival phase (i.e., early-phase, late-phase, and survivor). **(A)** ALT; **(B)** AST; **(C)** ALP; **(D)** GGT; **(E)** BUN; **(F)** CRE; **(G)** ALB; **(H)** AMY. Error bars represent the SEM.

**Figure 5:**
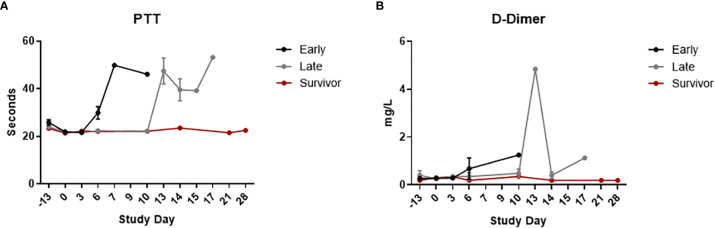
Coagulation. Coagulation parameters were measured using a Sysmex^®^ CA-1500 System. The graphs in this figure show data grouped by survival phase (i.e., early-phase, late-phase, and survivor). **(A)** PTT; **(B)** D-dimer. Error bars represent the SEM.

Clinical pathology data can be seen in **Figure 3** (hematology), **Figure 4** (clinical chemistry), and **Figure 5** (coagulation). For all clinical pathology analyses, changes are discussed in relation to baseline, which is defined as mean of data from Study Day -13 and Study Day 0 for a particular animal. With few exceptions and regardless of group, animals that did not survive had reductions in hematocrit (HCT) (with or without changes in other red blood cell parameters), reticulocytes (RETIC), and platelets (PLT), along with increases in white blood cells (WBC) and neutrophils (NEUT) counts. These findings indicate the presence of inflammation and/or hemorrhage (either active, resolving, or resolved). Large unstained cell (LUC) counts were also increased for a few animals; this change suggested a stress response and/or inflammation. Results of Wilcoxon rank-sum tests showed significant differences on Study Day 6 between early-phase and late-phase animals. WBC and NEUT percentages were significantly higher in the early-phase animals than in the late-phase animals, and PLT and LUC percentages were significantly lower in the early-phase animals than in the late-phase animals.

Decreased levels of albumin (ALB) (with or without decreases in other associated parameters, such as total protein and calcium), increased levels of BUN and CRE, increased and/or decreased levels of amylase (AMY), and increased levels of liver-related enzyme activities [alanine aminotransferase (ALT), aspartate aminotransferase (AST), alkaline phosphatase (ALP), and GGT] were commonly noted for non-survivors. A few of these animals also had increased levels of total bilirubin (TBIL), which may have been due, at least in part, to hepatocellular damage. ALB is a negative acute phase protein; decreases in this protein may have been due, at least in part, to inflammation and/or blood loss. Increases in BUN, CRE, and AMY may have been due, at least in part, to renal injury and/or pre-renal causes (such as dehydration). In addition, changes in the level of AMY in the blood (either increases or decreases) may have been due, at least in part, to pancreatic acinar cell damage. Results of Wilcoxon rank-sum tests showed significant differences on Study Day 6 between early-phase and late-phase animals. ALP and CRE were both significantly higher in the early-phase animals than in the late-phase animals, and AMY was significantly lower in the early-phase animals than in the late-phase animals.

Most non-survivors also had evidence of coagulopathy (increased clotting times and/or increased D dimers). Differences in magnitude of the D dimer response when late-phase animals were compared to early-phase animals were not considered significant as this observation was solely based on data from Study Day 13 which was only representative of one animal. Results of Wilcoxon rank-sum tests showed significant differences on Study Day 6 between the early-phase animals and the late-phase animals. Activated partial thromboplastin time was significantly higher in the early-phase animals than in the late-phase animals.

Similar to clinical disease signs, there was a rightward shift in the appearance of clinical pathology changes when late-phase and early-phase animals were compared, indicating a delay in disease development/progression. This delay in disease onset is the most likely explanation for the statistically-significant differences noted for clinical pathology parameters at Study Day 6 when early and late-phase animals were compared.

For the survivor, the only biologically relevant changes in clinical pathology parameters were increases in RETIC counts compared to baseline were noted, indicative of a regenerative response. The survivor also had a decrease in HCT and red blood cells that was accompanied by the increase in RETIC count, which likely indicated a regenerative response. WBC, NEUT, PLT, and LUC counts were not changed from baseline at any time point for this animal.

### Viral RNA Levels as Determined by qRT-PCR

**Figure 6:**
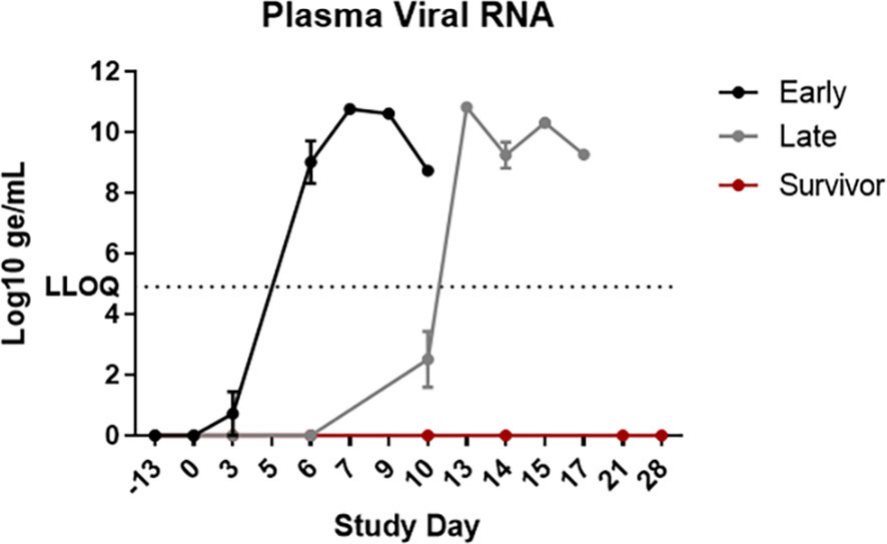
Viral RNA in EDTA plasma. EBOV-specific qRT-PCR was performed on RNA extracted from EDTA plasma. Data are shown as Log10 ge/mL. The graphs in this figure show data grouped by survival phase (i.e., early-phase, late-phase, and survivor). LLOQ, lower limit of quantification. Error bars represent the SEM.

Data from qRT-PCR analysis on plasma can be seen in **Figure 6**. Results of Wilcoxon rank-sum tests showed no significant differences in peak viral RNA levels between groups, or when early-phase animals were compared to late-phase animals, with group mean peak levels ranging from 8.63 Log_10_ genomic equivalents (ge)/mL to 10.57 Log_10_ ge/mL. However, a delay in the appearance of this disease sign was noted for late-phase animals. Whereas early-phase animals had detectable viral RNA in plasma by Study Day 6, viral RNA was not detected for late-phase animals until or after Study Day 10. There was no apparent difference in the time elapsed between detection and death when early and late-phase animals were compared, and peak RNA levels nearly always correlated with a terminal disease state. Viral RNA was not detected in the plasma at any time point for the end-of-study survivor.

### Gross, Histological and Molecular Pathology

A summary of the results for gross, histological, and immunohistochemical assessments can be found in **Tables 3**–**6**. The post-mortem lesions for all non-survivors were similar and consistent with EBOV disease in CM, and there were no appreciable differences when findings for groups were compared. Gross findings included maculopapular skin rashes and yellow-tan discoloration of the liver with/without increased friability. The most prominent histologic lesions in non-survivors were noted in the spleen and liver. In the spleen, findings included mild to severe lymphoid depletion, lymphocytolysis, expansion of the red pulp with fibrin, and hemorrhage and/or congestion within the follicles and perifollicular areas (marginal zones). In the liver, findings included minimal to moderate degeneration and necrosis of individual hepatocytes with varying amounts of inflammation. Nasal sections (nasal septum and turbinate bones with mucosal epithelium) had one or more of the following microscopic changes for all non-survivors: inflammation, vascular congestion and/or hemorrhage, intravascular fibrin thrombi. Less frequently, non-survivors had lymphoid depletion within the lymph nodes, as well as hepatocellular degeneration and necrosis with or without inflammation. Of the lymph nodes, the axillary, tracheobronchial and mesenteric nodes were, for most part, similarly affected. In non-survivors, lymphoid depletion with lymphocytolysis and infiltration of the sinuses by histiocytes predominated. Minimal adrenal gland degeneration and necrosis, and minimal inflammation within the adrenal gland were noted for a few animals and are, therefore, not considered prominent or characteristic findings for this model. With the exception of gastrointestinal (GI) lesions, appreciable differences in findings between early and late-phase animals were not noted. However, GI lesions, when present, were predominantly noted for late-phase animals.

**Table 3:** Summary of gross pathological findings.

	CM #	1	2	3	4	5	6	7	8	9	10	11	12	13	14	15	16	17	18	19	20	21
	Group	1	1	1	1	1	1	2	2	2	2	2	2	3	3	3	3	3	4	4	4	4
	Phase	Early	Early	Late	Early	Late	Late	Early	Late	Late	Late	EOS	Late	Late	Early	Late	Early	Late	Early	Early	Early	Late
Skin	Petechial or macular rash	**(+)**	**(+)**	**(+)**	**(+)**	**(+)**	**(+)**	**(+)**	**(+)**		**(+)**		**(+)**	**(+)**	**(+)**	**(+)**	**(+)**	**(+)**	**(+)**	**(+)**	**(+)**	
Lung	Nodule																					
TB LN	Enlarged and/or discolored							**(+)**										**(+)**				
Adrenal gland	Hemorrhage		**(+)**	**(+)**			**(+)**			**(+)**	**(+)**			**(+)**								**(+)**
	Friable			**(+)**										**(+)**								
Liver	Friable	**(+)**	**(+)**	**(+)**	**(+)**	**(+)**	**(+)**	**(+)**	**(+)**		**(+)**		**(+)**	**(+)**	**(+)**		**(+)**	**(+)**	**(+)**	**(+)**		**(+)**
	Discolored	**(+)**	**(+)**	**(+)**	**(+)**	**(+)**	**(+)**	**(+)**	**(+)**		**(+)**		**(+)**	**(+)**	**(+)**		**(+)**	**(+)**	**(+)**	**(+)**		**(+)**
Kidney	Pale				**(+)**																	
Body condition	Obese		**(+)**	**(+)**	**(+)**		**(+)**		**(+)**	**(+)**				**(+)**				**(+)**		**(+)**		
	Scant adipose tissue										**(+)**	**(+)**										
	Fluid in body cavities												**(+)**		**(+)**		**(+)**					
GI tract	Ulcer(s)							**(+)**														
	Mass/nodule								**(+)**													
	Dark red (hemorrhagic)						**(+)**	**(+)**	**(+)**	**(+)**			**(+)**									**(+)**
	Pin point hemorrhages						**(+)**			**(+)**											**(+)**	**(+)**
	Discoloration													**(+)**						**(+)**		
Testicles	Reddened (hemorrhage)					**(+)**		**(+)**							**(+)**	**(+)**	**(+)**		**(+)**			**(+)**

+, indicates the presence of the gross lesion; no result, absence.

EOS, end of study (survivor).

**Table 4:** Summary of histological findings.

	CM #	1	2	3	4	5	6	7	8	9	10	11	12	13	14	15	16	17	18	19	20	21
	Group	1	1	1	1	1	1	2	2	2	2	2	2	3	3	3	3	3	4	4	4	4
	Phase	Early	Early	Late	Early	Late	Late	Early	Late	Late	Late	EOS	Late	Late	Early	Late	Early	Late	Early	Early	Early	Late
Liver	Hepatocellular degeneration and necrosis	**(+)**	**(+)**	**(+)**	**(+)**	**(+)**		**(+)**	**(+)**	**(+)**	**(+)**		**(+)**	**(+)**	**(+)**	**(+)**	**(+)**	**(+)**	**(+)**	**(+)**	**(+)**	
	Inflammation			**(+)**	**(+)**		**(+)**			**(+)**			**(+)**					**(+)**				
Kidney	Tubular degeneration and necrosis	**(+)**	**(+)**	**(+)**	**(+)**	**(+)**	**(+)**	**(+)**		NE	**(+)**		**(+)**	**(+)**		NE	NE	**(+)**	NE	**(+)**	NE	NE
	Fibrin thrombi	**(+)**	**(+)**			**(+)**		**(+)**	**(+)**	NE	**(+)**		**(+)**	**(+)**		NE	NE	**(+)**	NE	**(+)**	NE	NE
	Inflammation				**(+)**								**(+)**			NE	NE	**(+)**	NE		NE	NE
	Tubular casts		**(+)**		**(+)**	**(+)**	**(+)**	**(+)**	**(+)**	NE	**(+)**		**(+)**			NE	NE	**(+)**	NE	**(+)**	NE	NE
	Mineral deposits			**(+)**			**(+)**		**(+)**	NE			**(+)**	**(+)**		NE	NE	**(+)**	NE	**(+)**	NE	NE
GI Tract	Inflammation	**(+)**	**(+)**	**(+)**	**(+)**	**(+)**	**(+)**			**(+)**	**(+)**	**(+)**	**(+)**	**(+)**	NE	**(+)**	**(+)**	**(+)**	**(+)**	**(+)**		**(+)**
	Degeneration and necrosis		**(+)**		**(+)**	**(+)**	**(+)**			**(+)**	**(+)**				NE				**(+)**			**(+)**
	Fibrin thrombi	**(+)**	**(+)**			**(+)**		**(+)**	**(+)**	**(+)**	**(+)**		**(+)**		NE	**(+)**	**(+)**		**(+)**	**(+)**		**(+)**
	Necrotic/apoptotic debris		**(+)**	**(+)**	**(+)**			**(+)**	**(+)**						NE		**(+)**	**(+)**	**(+)**	**(+)**		**(+)**
	Hemorrhage						**(+)**	**(+)**	**(+)**	**(+)**			**(+)**	**(+)**	NE							**(+)**
Nasal Sections	Inflammation	**(+)**	**(+)**	NE	**(+)**	**(+)**	**(+)**	**(+)**	**(+)**	**(+)**	**(+)**	**(+)**	**(+)**	NE	**(+)**	**(+)**	**(+)**	**(+)**	**(+)**	**(+)**	NE	
	Congestion/hemorrhage		**(+)**	NE		**(+)**	**(+)**	**(+)**					**(+)**	NE						**(+)**	NE	
	Fibrin thrombi	**(+)**	**(+)**	NE	**(+)**	**(+)**	**(+)**		**(+)**	**(+)**	**(+)**		**(+)**	NE	**(+)**	**(+)**	**(+)**	**(+)**	**(+)**	**(+)**	NE	**(+)**
Lung	Interstitial inflammation		**(+)**	**(+)**			**(+)**				**(+)**				**(+)**	**(+)**	**(+)**	**(+)**	**(+)**			
	Pleuritis		**(+)**																**(+)**			
	Fibrin thrombi					**(+)**										**(+)**						
Gonad	Inflammation		**(+)**	**(+)**	**(+)**	**(+)**			**(+)**			**(+)**	**(+)**					**(+)**		**(+)**		
Adrenal Gland	Inflammation					**(+)**																
	Degeneration and necrosis							**(+)**										**(+)**		**(+)**		

+, indicates the presence of the gross lesion, no result = absence.

EOS, end of study (survivor).

NE, Not evaluated.

**Table 5:** Summary of histological findings – lymphoid tissues.

	CM #	1	2	3	4	5	6	7	8	9	10	11	12	13	14	15	16	17	18	19	20	21
	Group	1	1	1	1	1	1	2	2	2	2	2	2	3	3	3	3	3	4	4	4	4
	Phase	Early	Early	Late	Early	Late	Late	Early	Late	Late	Late	EOS	Late	Late	Early	Late	Early	Late	Early	Early	Early	Late
Spleen	Lymphoid depletion	**(+)**	**(+)**	**(+)**	**(+)**	**(+)**	**(+)**	**(+)**	**(+)**	**(+)**	**(+)**		**(+)**	**(+)**	**(+)**	**(+)**	**(+)**	**(+)**	**(+)**	**(+)**	**(+)**	**(+)**
	Hemorrhage/congestion	**(+)**	**(+)**	**(+)**	**(+)**	**(+)**	**(+)**	**(+)**	**(+)**	**(+)**	**(+)**		**(+)**	**(+)**	**(+)**	**(+)**	**(+)**	**(+)**	**(+)**	**(+)**	**(+)**	**(+)**
	Red pulp, fibrin	**(+)**	**(+)**	**(+)**	**(+)**	**(+)**	**(+)**	**(+)**	**(+)**	**(+)**	**(+)**		**(+)**	**(+)**	**(+)**	**(+)**	**(+)**	**(+)**	**(+)**	**(+)**	**(+)**	**(+)**
	Necrotic/apoptotic debris	**(+)**	**(+)**	**(+)**	**(+)**	**(+)**	**(+)**	**(+)**	**(+)**	**(+)**	**(+)**		**(+)**	**(+)**	**(+)**	**(+)**	**(+)**	**(+)**	**(+)**	**(+)**	**(+)**	**(+)**
	Lymphoid hyperplasia											**(+)**										
Axillary LN	Lymphoid depletion	**(+)**	**(+)**	NE	**(+)**	**(+)**	**(+)**	**(+)**		**(+)**	**(+)**		**(+)**		**(+)**	**(+)**	**(+)**		**(+)**	**(+)**		**(+)**
	Sinus histiocytosis	**(+)**		**(+)**	**(+)**	**(+)**	**(+)**	**(+)**	**(+)**	**(+)**	**(+)**	**(+)**		**(+)**	**(+)**	**(+)**	**(+)**	**(+)**	**(+)**	**(+)**	**(+)**	
	Fibrin thrombi/vascular necrosis					**(+)**		**(+)**			**(+)**				**(+)**	**(+)**				**(+)**		
	Lymphoid hyperplasia											**(+)**										
Mesenteric LN	Lymphoid depletion	**(+)**	**(+)**	NE	**(+)**	**(+)**	**(+)**	**(+)**	**(+)**	NE	**(+)**		**(+)**	**(+)**	**(+)**	**(+)**	**(+)**	**(+)**	**(+)**	**(+)**	**(+)**	**(+)**
	Sinus histiocytosis	**(+)**	**(+)**	**(+)**	**(+)**	**(+)**		**(+)**	**(+)**	**(+)**	**(+)**	**(+)**	**(+)**	**(+)**	**(+)**	**(+)**	**(+)**	**(+)**	**(+)**	**(+)**	**(+)**	
	Fibrin thrombi/vascular necrosis		**(+)**				**(+)**	**(+)**			**(+)**		**(+)**									
	Lymphoid hyperplasia											**(+)**										
Mediastinal LN	Lymphoid depletion	**(+)**	**(+)**	**(+)**	**(+)**	**(+)**	**(+)**	**(+)**	**(+)**	**(+)**	**(+)**		**(+)**	**(+)**	**(+)**	**(+)**	**(+)**	**(+)**	NE	**(+)**	**(+)**	NE
	Sinus histiocytosis	**(+)**			**(+)**	**(+)**			**(+)**		**(+)**	**(+)**	**(+)**		**(+)**		**(+)**	**(+)**		**(+)**	**(+)**	
	Fibrin thrombi/vascular necrosis	**(+)**	**(+)**	**(+)**			**(+)**	**(+)**		**(+)**	**(+)**			**(+)**	**(+)**	**(+)**		**(+)**		**(+)**	**(+)**	**(+)**
	Lymphoid hyperplasia											**(+)**										
Thymus	Lymphoid depletion	**(+)**	**(+)**					**(+)**			**(+)**		**(+)**				**(+)**		**(+)**	**(+)**	**(+)**	**(+)**
	Lymphoid atrophy	**(+)**	**(+)**								**(+)**		**(+)**				**(+)**		**(+)**	**(+)**	**(+)**	**(+)**

+, indicates the presence of the gross lesion, no result = absence.

EOS, end of study (survivor).

NE, Not evaluated.

**Table 6:** Summary of immunohistochemical findings.

CM #	1	2	3	4	5	6	7	8	9	10	11	12	13	14	15	16	17	18	19	20	21
Group	1	1	1	1	1	1	2	2	2	2	2	2	3	3	3	3	3	4	4	4	4
Phase	Early	Early	Late	Early	Late	Late	Early	Late	Late	Late	EOS	Late	Late	Early	Late	Early	Late	Early	Early	Early	Late
Lung	2	3	2	2	2	3	3	1	2	2	0	2	1	2	2	2	2	3	3	2	2
Liver	4	3	2	2	3	2	4	5	4	4	0	1	5	2	3	3	2	3	5	2	2
Nares	1	0	2	1	1	1	2	0	0	0	0	0	0	1	0	1	1	0	2	0	1
Trachea	1	1	2	1	2	1	1	1	1	1	0	1	2	1	1	1	2	1	2	2	2
Gonad	2	2	3	3	2	2	1	3	2	1	0	2	4	2	1	2	3	2	4	1	2
GI Tract	3	3	3	2	2	2	3	3	3	2	0	3	2	3	2	3	2	2	3	2	3
Adrenal Gland	2	2	2	2	2	2	3	3	2	3	0	2	4	2	2	2	2	1	3	2	2

0 = not immunoreactive; no cells in the section are immunoreactive.

1 = minimal, up to 10% of the cells in the section are immunoreactive.

2 = mild, 11-25% of the cells in the section are immunoreactive.

3 = moderate, 26-50% of the cells in the section are immunoreactive.

4 = marked, 51-79% of the cells in the section are immunoreactive.

5 = severe, 80% or more of the cells in the section are immunoreactive.

EOS, end of study (survivor).

EBOV antigen was detected by immunohistochemistry in all CMs with histologic evidence of an active EBOV infection. Immunoreactivity in the lung, liver, trachea, gonad, GI tract, and adrenal gland was similar within the non-survivors and consistent with EBOV disease in CM. There were no appreciable differences when immunoreactivity of these tissues were compared between study groups and between early and late-phase animals.

Although the majority of critical-phase animals had measurable immunoreactivity within their nares, immunoreactivity in the nares was only consistently observed for late-phase animals in Group 1 (the 10 pfu drop-wise group). This variability in immunoreactivity could be attributed to an uneven presence of host-derived local mucosal (IgA) antibody, which was blocking antigen binding sites, a difference in viral particle dispersion between the two viral delivery devices for the 10 pfu group, and/or an absence or cessation of viral replication in the nares for late-phase animals.

The only gross finding for the end-of-study survivor was scant visceral and subcutaneous adipose tissue. Histologic findings for the survivor included expansion of the white pulp of the spleen by lymphoid hyperplasia (most frequently within the germinal centers of the splenic nodules), lymphoid expansion and increased numbers of lymphoid follicles in lymph nodes, and inflammation in nasal sections, the GI tract, and the gonad. No evidence of viral antigen was found in all examined tissues for this animal.

## Discussion

EBOV remains a significant public health concern due to high morbidity and mortality rates during recurrent outbreaks in endemic areas, particularly Central Africa. More recent outbreaks in West Africa have demonstrated the ability of this virus to cross borders and cause severe disease and death in naïve populations. Therefore, the development of countermeasures against EBOV is a high priority, and requires the availability of appropriate animal models for efficacy evaluations. The objective of this study was to evaluate the EBOV disease course that arises after CMs are exposed to EBOV by the IN route. This exposure route is relevant because it mimics a common natural disease transmission route, which is predicted to be mucosal (4–6). Two different doses (10 pfu and 100 pfu) were used on this study to evaluate dose-dependent differences in pathogenesis. In addition, two delivery methodologies (drop-wise and MAD) were employed to evaluate which mechanism resulted in the most consistent disease among animals in a dose group.

In general and regardless of study group and delivery methodology, EBOV disease findings for all non-survivors were similar and consistent with historical EBOV-challenged CMs (9–12) and human cases (13, 14). Non-surviving animals on this study were also grouped based on the number of days post-exposure they reached a terminal disease state, with animals in the “early-phase” group reaching terminal disease between Study Day 6 and Study Day 10, and animals in the “late-phase” group reaching terminal disease between Study Day 12 and Study Day 18. The mean difference in time to death between early-phase and late-phase groups was approximately 6 days. The timing of appearance EBOV disease manifestations (i.e., viral RNA in plasma, clinical signs, fever, and clinical pathology aberrations) was also different between early and late-phase animals, with manifestations for late-phase animals appearing on or after Study Day 10. The data, therefore, support the conclusion that there were two distinct critical phases on this study, and approximately half of the animals in each study group were distributed between each critical phase. In addition, the time to death for critical-phase animals infected with the target dose of 100 pfu EBOV was also approximately 1.7 days longer than CMs infected with EBOV by the IM or AE routes (9–12). Typically, CMs infected with EBOV by the IM or AE route reach terminal disease between Study Day 6 and Study Day 8, whereas 75 percent (three out of four) of 100 pfu early-phase animals on this study reached terminal disease on Study Day 9 and Study Day 10. This observation, in association with the time to death for 100 pfu late-phase animals, suggests a delayed disease course when CMs are infected with EBOV by the IN route. Although the reason for this delayed disease, which is not seen for IM or AE routes of infection, was not investigated on this study, we might speculate that the initial target cells during an intranasal instillation, which are likely upper respiratory tract tissues, are less permissible to sustained virus infection compared to lower respiratory tract cells or muscle tissue. This could be associated with reduced replication capacity leading to a slower disease progression. However, this prediction is highly speculative and warrants further investigation. It should be noted that we did not investigate the effect of higher doses (i.e., 10^3^ pfu, 10^4^ pfu, etc.), or the use of other variants (notably an 8U variant), on this study. Whether delayed disease would still be seen after modifying these variables is unknown and also warrants further investigation.

There were a few notable differences in observed findings for early-phase and late-phase animals, and these included weight loss, gross GI lesions, and immunoreactivity in nare tissue. Weight loss, when noted, was more often associated with late-phase animals. The difference in weight loss cannot simply be explained as a function of EBOV disease, as late-phase animals exhibited clinical disease signs for the same duration (from onset to death) as early-phase animals. Weight loss for late-phase animals may have been due, at least in part, to a stress response; however, an exact cause cannot be gleaned from the information produced by this study, and further work is necessary to determine if this finding is consistent across studies or was coincidental.

Gross GI lesions, when noted, were more often associated with late-phase animals. However, histologic GI findings did not show the same distribution, with at least one finding in the GI tract noted for all early-phase animals and the majority of late-phase animals. Therefore, the observation that gross GI lesions were more often noted for late-phase animals is likely not of significance.

Immunoreactivity in the nares, when noted, was more often associated with early-phase animals and 10 pfu drop-wise late-phase animals. This variability in immunoreactivity could be attributed to an uneven presence of host-derived local mucosal (IgA) antibody which was blocking antigen binding sites, a difference in viral particle dispersion between the two viral delivery devices for the 10 pfu group, and/or to an absence or cessation of viral replication in the nares for late-phase animals.

The few findings for the survivor did indicate this animal was likely infected with EBOV; however, this animal did not develop severe clinical disease, and antibody assays were not conducted on this study to demonstrate seroconversion for this animal. Clinically, lymphadenopathy was noted for this animal during the typical EBOV critical phase period (i.e., between Study Day 6 and Study Day 10). Hematologically, findings were indicative of a regenerative response. Pathologically, expansion of the white pulp of the spleen by lymphoid hyperplasia (most frequently within the germinal centers of the splenic nodules), lymphoid expansion and increased numbers of lymphoid follicles in lymph nodes, and inflammation in nasal sections, the GI tract, and the gonad suggested the presence of prior viral infection that had been cleared at the time of necropsy. This animal was in a 10 pfu dose group, suggesting it might be possible to achieve a sub-lethal model of EBOV using the IN route of exposure by reducing the exposure dose below 10 pfu; however, to date, this has not been attempted.

A manuscript published in 2017 described the development of the first lethal IN EBOV model in CMs (7). Similar to survival information provided in this manuscript for early-phase animals, Alfson et al. observed a statistically significant increase in time to death for CMs infected by the IN route with a target dose of either 120 or 500 pfu EBOV (mean = 8.9 days) compared to animals infected by the IM route with a target dose of 100 pfu EBOV (mean = 7 days). However, a group of animals analogous to the late-phase animals described herein were not noted in the published study, and there were no survivors in the published study. Late-phase animals represented a significant proportion of the non-surviving animals on the present study (12 out of 20, or 60%), demonstrating that this group was not coincidental. In addition, a similar disease phenomenon was noted in CMs infected with the Makona variant of EBOV; in that study, 7 out of 10 non-surviving animals (70%) reached terminal disease after Study Day 10, and two animals survived until end-of-study (14). Alfson et al. also described a prolonged clinical disease from onset to death for IN animals compared to IM animals. This was not observed in the present study as there were two distinct critical phases, and the time from clinical disease onset to death was similar when IN animals were compared to historic IM and AE animals (9–12). The reason for the discrepancy between the Alfson et al. study and the present study in terms of late-phase animals is unclear but could be related, at least in part, to smaller group sizes in the Alfson et al. study, animal origin differences (Chinese for the present study versus Vietnamese for the Alfson et al. study), and/or differences in the euthanasia criteria employed.

The IM model of EBOV disease in the CM was developed to mimic an exposure through needle stick, such as might occur for laboratory personnel or health care workers. However, this model has a shortened disease course compared to human disease, does not allow for evaluation of viral persistence as survivors are not obtained following IM EBOV exposure in CMs, and does not represent a predicted natural route of infection. Other disease characteristics of human EBOV are evident in the CM IM model, including (but not limited to), fever, maculopapular rash, GI involvement, hemorrhage, high viremia, elevated liver enzymes, azotemia, and coagulopathy (decreases platelets and increased clotting times) (9–12). Mucosal routes of exposure to EBOV are predicted for human outbreaks (3–6), and the intranasal model described in this report and in the Alfson et al. study (7) has been investigated as a viable mucosal model candidate in CMs. Similar to the IM model, clinical disease characteristics of the IN model closely mimic those seen in human EBOV cases. However, unlike the traditional IM model (which uses between 100 and 1,000 pfu as a target inoculation dose), the IN model more accurately replicates a human disease course. In addition, the potential for survivors opens the possibility that long-term viral persistence and sequelae may be examined using this model. In summary, the data presented support the need for further development of this model as a possible alternative to parenteral models for the study of human EBOV disease and for countermeasure evaluations.

## Data Availability Statement

The original contributions presented in the study are included in the article/supplementary material. Further inquiries can be directed to the corresponding authors.

## Ethics Statement

The animal study was reviewed and approved by Institutional Animal Care and Use Committee - USAMRIID.

## Author Contributions

SJ and AG designed the study. SJ, JS, AK, JZ, and SW performed study observations and data collection. CW performed pathological evaluations and analyses. SJ performed data compilation and analysis. SJ drafted the manuscript. AG performed manuscript review. All authors contributed to the article and approved the submitted version.

## Funding

Funding for this effort was provided by the National Institutes of Allergy and Infectious Diseases under project number 188104641.

## Conflict of Interest

The authors declare that the research was conducted in the absence of any commercial or financial relationships that could be construed as a potential conflict of interest.

## Publisher’s Note

All claims expressed in this article are solely those of the authors and do not necessarily represent those of their affiliated organizations, or those of the publisher, the editors and the reviewers. Any product that may be evaluated in this article, or claim that may be made by its manufacturer, is not guaranteed or endorsed by the publisher.
